# Association of Bovine Respiratory Disease during the Pre-Weaning Period with Blood Cell Counts and Circulating Concentration of Metabolites, Minerals, and Acute Phase Proteins in Dairy Calves Transported to a Calf Raising Facility

**DOI:** 10.3390/ani14131909

**Published:** 2024-06-28

**Authors:** Lauren Paige Bielamowicz, Maria Luiza Celestino, Paulo R. Menta, Leticia Fernandes, Michael Ballou, Rafael C. Neves, Vinicius S. Machado

**Affiliations:** 1 Department of Veterinary Sciences, Davis College of Agricultural Sciences and Natural Resources, Texas Tech University, Lubbock, TX 79409, USA; 2 Department of Veterinary Clinical Sciences, College of Veterinary Medicine, Purdue University, West Lafayette, IN 47907, USA

**Keywords:** transportation stress, haptoglobin, bovine respiratory disease, white blood cells, calves

## Abstract

**Simple Summary:**

Dairy calves are often subjected to transportation at a young age and this may increase their risk of bovine respiratory disease (BRD). Many previous investigations evaluated the association of BRD with blood cells and with the concentration of metabolites, minerals, and proteins associated with an inflammatory response. However, these factors can also be altered by transportation stress, and information regarding how these variables are associated with BRD in calves that undergo transportation stress in early life is limited. This study aimed to evaluate the association of BRD with several variables related to biomarkers of metabolism and inflammation in dairy calves transported to a calf raising facility within the first few days of life. Blood was collected at 7, 17, 34, and 56 days of age, and several variables were associated with BRD. For instance, calves diagnosed with BRD within their first 30 days had elevated haptoglobin, a biomarker of inflammation, at 7 days of age, but at 56 days of age, the concentration of this biomarker was reduced in comparison to healthy calves. Our findings demonstrate how these variables are associated with BRD after calves undergo long distance transportation.

**Abstract:**

Our objective was to investigate the association of bovine respiratory disease (BRD) occurring within the first 56 days of life with blood cell counts and the circulating concentration of metabolites, minerals, and acute phase proteins throughout the pre-weaning period in dairy calves transported to a heifer raising facility within their first week of life. Data from 305 calves transported from dairies in Minnesota to a calf raising facility in New Mexico within their first four days of life were used in this retrospective cohort study. Blood samples were collected at 7, 17, 34, and 56 days of life for the analysis of blood cell counts, biochemistry, and the concentration of acute phase proteins. Blood urea nitrogen, albumin, GLDH, CK, P, Na, K, Cl, Zn, Hp, SAA, and monocyte counts were associated with BRD status throughout or at least at one of the time points evaluated in this study. In conclusion, several hematological variables were associated with BRD status in dairy calves that underwent transportation stress in early life.

## 1. Introduction

Bovine respiratory disease (BRD) is a multifactorial disease characterized by an upper respiratory tract infection that causes significant morbidity and mortality in pre-weaned dairy calves [1,2]. Bovine respiratory disease can have a bacterial or viral etiology, and it is associated with pathogens such as *Mannheimia haemolytica*, *Pasteurella multocida*, *Mycoplasma bovis*, bovine viral diarrhea virus, bovine respiratory syncytial virus, parainfluenza virus-3, bovine herpes virus-1 and multiple others [3]. Bovine respiratory disease is diagnosed by assessment of nasal and eye discharge, ear droop, head tilt, coughing, breathing, and rectal temperature [4]. In addition to the detrimental impact of BRD on welfare and growth performance during the pre-weaning period, affected calves display long-term impacts such as reduced growth after weaning, impaired fertility, and decreased milk production during the first lactation [5,6]. Moreover, calves diagnosed with BRD are less likely to reach lactation due to high mortality and cull rates [5,7].

The risk of BRD is increased when stressors like transportation are added to calves’ lives [8]. In fact, many dairy calves confront a long-distance trip to an off-site calf raising facility at a very young age [9]. At this early stage of life, the calves’ immunity is not fully established as they heavily rely on maternal antibodies to protect against pathogens [10]. This further contributes to increased BRD risk when combined with stressors such as transportation and comingling [6,10]. Transportation has been reported to lead to physiological changes and immunosuppression that increase the calves’ susceptibility to pathogens [11,12,13,14]. For instance, mineral metabolism [6] and hematological [14,15] changes have been observed after transportation of calves. Additionally, transportation of calves was recently reported to alter the circulating concentrations of acute phase proteins, such as haptoglobin (Hp) and serum amyloid A (SAA) [13,14]. Many of these variables that are altered during and after stressful events such as transportation and weaning are also indicative of disease [16]. For instance, an elevated neutrophil to lymphocyte ratio or the concentration of acute phase proteins were associated with BRD in calves [16,17], indicating that these variables can be important biomarkers of BRD and may also be used to aid in the diagnosis of diseases in calves [18].

Most of the research demonstrating the association of BRD with the dynamics of several biomarkers of immunity, inflammation, and metabolism during the pre-weaning period of dairy calves have been conducted in conditions without the added stress caused by early life long-distance transportation. Hence, the objective of the study was to evaluate the association between BRD and multiple circulating biomarkers measured at different times throughout the pre-weaning period of calves that were transported to an off-site calf raising facility within the first week of life. Our hypothesis was that blood cell counts and the blood concentration of several metabolites, minerals, and acute phase proteins differed between calves with and without BRD during the pre-weaning period.

## 2. Materials and Methods

All activities performed in this study were reviewed and approved by the Texas Tech University Institutional Animal Care and Use Committee (#18081-10).

### 2.1. Study Animals, Farm Management, and Inclusion Criteria

Data from a random subset of 305 Jersey and Jersey-cross heifer calves from a total of 2100 calves enrolled in a study evaluating the efficacy of metaphylactic strategies to prevent and control BRD in long-distance transported calves [19] were used for the present study. Calves originated from 13 dairy farms in Minnesota (MN), where they were fed 4 L of pasteurized pooled colostrum within the first 6 h of life and received subsequent feedings of 1.8 L of milk replacer (27% crude protein, 25% crude fat, DM basis) twice daily. Within the first week of life, the calves were transported to a calf raising facility located in New Mexico (NM). The transportation distance was approximately 1715 km and its use is part of the regular routine of the participating farms and the calf ranch. After arrival, the calves were raised in individual hutches, whole milk was fed twice a day (4 L/d), and water and calf starter were offered ad libitum throughout the pre-weaning period. Additionally, calves received intranasal immunization against BRSV, IBR, and PI3 (Inforce 3, Zoetis, MI, USA) at birth, and were vaccinated with a *Mannheimia haemolytica* bacterin-toxoid bacteria (One Shot, Zoetis, MI, USA) at 30 days of age and at weaning.

In the clinical trial, 2100 calves were randomly allocated into three treatment groups. Briefly, enrollment was based on testing negative for BVD and not presenting clinical signs associated with BRD (nasal or ocular discharge, cough, ear droop, and rectal temperature ≥39.2 °C). To test for BVD, fresh skin samples collected at enrollment were submitted to the Texas A&M Veterinary Medical Diagnostic Laboratory in Amarillo, TX, USA for antigen capture using an ELISA method. Calves enrolled in the META1 group received a single subcutaneous injection of tildipirosin (Zuprevo™, Parsippany-Troy Hills, NJ, USA, Merck Animal Health, Rahway, NJ, USA; 4 mg/kg of body weight) at enrollment. Calves in group META2 received two subcutaneous injections of tildipirosin (4 mg/kg of body weight) 17 days apart. Calves in the CON group remained as untreated controls. Calves were enrolled in the study three days after arrival at the NM calf raising facility. To evaluate the effect of metaphylaxis on biomarkers of metabolism and inflammation, blood was collected from a random subset of 305 calves. Randomization was performed using the random number function of Excel (Microsoft Corp., Redmond, WA, USA). Eligibility to be included in the present study was based on the availability of the blood measurement data.

### 2.2. Data Collection and Bovine Respiratory Disease Case Definition

From enrollment (approximately 7 days of age) until weaning (approximately 56 days of age), calves were visually inspected 3 times a week (on a Monday-Wednesday-Friday basis) by the research crew. Using a systematic scoring system [4], calves were screened for BRD based on the presence of nasal discharge (4 points), ocular discharge (2 points), abnormal respiration (2 points), cough (2 points), and ear droop or head tilt (5 points). If calves had a score = 4, then rectal temperature was assessed (rectal temperature ≥ 39.2 °C = 2 points). Bovine respiratory disease was characterized as a score ≥5, and calves diagnosed with BRD were immediately treated with 40 mg/kg florfenicol and 2.2 mg/kg flunixin meglumine (Resflor Gold^®^, Merck Animal Health, Rahway, NJ, USA). Calves were re-treated with a different drug class (e.g., Enrofloxacin, Baytril^®^ 100, Bayer, Whippany, NJ, USA) if the clinical signs of BRD persisted four days after initial diagnosis. Animals enrolled in META1 and META2 had a 3-day post metaphylactic interval when they were not eligible to receive subsequent antimicrobial therapy.

Body weight was assessed at enrollment using a digital scale (Calf Cart™, Raytec^®^, Ephrata, PA, USA), while the rectal temperature at enrollment was measured using a digital thermometer (GLA M900, GLA Agriculture Electronics, San Luis Obispo CA, USA) equipped with a 10 cm angle probe. Data related to date of birth, dam’s parity, dam’s gestation length and source (farm of origin) were collected from the farm’s database software (DairyComp 305, Valley Agricultural Software, https://vas.com/, Tulare, CA, USA).

### 2.3. Blood Collection and Analysis

Blood samples were collected at approximately 7, 17, 34, and 56 days of age through the jugular vein using a Vacutainer tube without anticoagulant and a Vacutainer tube with EDTA, and a 20-gauge ×2.54-cm Vacutainer needle (Becton, Dickinson and Company, Franklin Lakes, NJ, USA). Blood samples were transported to the laboratory on ice and were processed or analyzed within 2 h after collection. Complete blood cell (CBC) counts and differentials were performed on the blood samples with EDTA, using a hematology analyzer (IDEXX Procyte DX, Westbrook, ME, USA). Serum was harvested from the blood samples without anticoagulant after centrifugation at 2000× *g* for 15 min at 4 °C, and frozen at −80 °C until further analyses were conducted.

For the analysis of total protein, albumin, calcium, phosphorus, glucose, blood urea nitrogen (BUN), creatine kinase, bilirubin, creatinine kinase (CK), aspartate aminotransferase (AST), globulins, gamma-glutamyl transferase (GGT), glutamate dehydrogenase activity (GLDH), magnesium, sodium, potassium, and chloride, a 0.5 mL aliquot of serum was submitted to the Texas A&M Veterinary Medical Diagnostic Laboratory for a ruminant chemistry profile. Measurements of the circulating concentration of insulin were performed using a commercial kit following the manufacturer’s instructions (Bovine Insulin ELISA, ALPCO, Salem, NH, USA). The circulating serum amyloid A (SAA) concentration was determined by a commercially available ELISA kit following the manufacturer’s instructions (Life Diagnostics, West Chester, PA, USA). The intra- and inter-assay coefficients of variation (CV) were <5.5 and <7.5%, respectively. The serum zinc concentration was determined using a chemistry analyzer (RX Daytona; RANDOX Laboratories, Crumlin, UK) in a single assay (intra-assay CV was 1.9%). Serum haptoglobin (Hp) concentration was determined using a colorimetric assay via quantification of the haptoglobin/hemoglobin complex by the estimation of differences in peroxidase activity [20], and this methodology was previously described in detail [19].

### 2.4. Statistical Analysis

The distribution of BRD cases throughout the pre-weaning period was plotted using the distribution plot function in MedCalc version 20.027 software (MedCalc Software, Mariakerke, Belgium). To account for the time when BRD occurred during the pre-weaning period, the variable BRD status was created based on days of age when BRD was diagnosed (NBRD = no BRD; EBRD = BRD diagnosed ≤ 30 days of age; LBRD = BRD diagnosed > 30 days of age). Descriptive statistics were calculated using the chi-square and ANOVA functions of JMP Pro16 (SAS Institute Inc., Cary, NC, USA). Several mixed linear models were fitted to the data using the MIXED procedure of SAS (SAS Institute Inc.) to assess the association between BRD status and blood biomarkers of metabolism and inflammation throughout the pre-weaning period. The data comprised a series of repeated measures of each dependent variable throughout the four blood collection days. To account appropriately for within-calf correlations, the error term was modeled by imposing a heterogenous autoregressive covariance structure for all models. The normality of the residuals was analyzed with normal probability and box plots visualization. If the normality of residuals criteria was not met, the dependent variable was either log or square root transformed.

For all models described above, independent variables and their respective interactions were kept when *p* < 0.10. The effect of BRD status, time, and their interaction were forced into all statistical models even in the absence of statistical significance. The SLICE option using a Tukey–Kramer multiple comparison adjustment was used to explore interactions between treatment and time whenever *p* < 0.10. Age in days at enrollment, body weight at enrollment, dam’s parity (lactation 1, lactation 2 or lactation ≥2), season (winter or spring), and rectal temperature at enrollment were offered to all models. Farm of origin (source) and treatment were included as random variables in all models. Statistical significance was considered at *p* < 0.05.

## 3. Results

### 3.1. Descriptive Statistics

**Figure 1:**
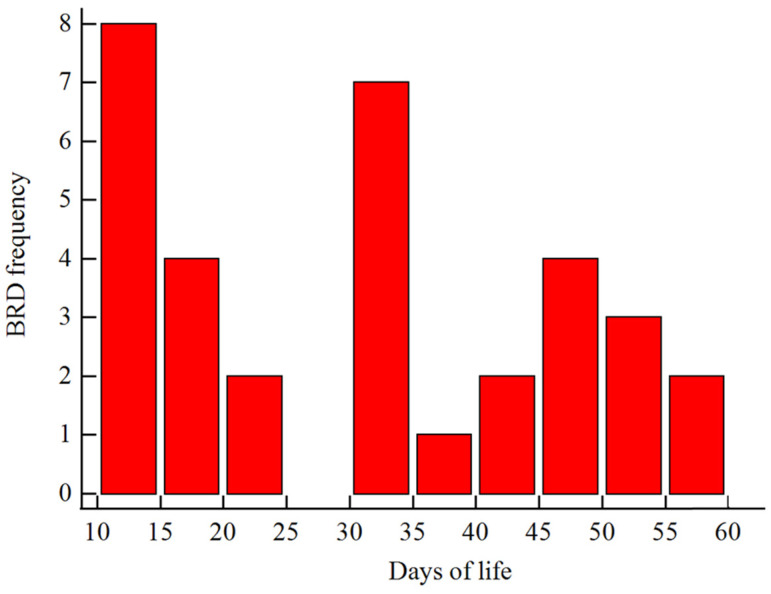
Distribution plot of bovine respiratory disease (BRD) incidence curve during the pre-weaning period.

The incidence of BRD in the study cohort was 11.1%. The incidence curve of BRD during the pre-weaning period is presented in Figure 1. A total of 271, 16, and 18 calves were enrolled in NBRD, EBRD, and LBRD, respectively.

The descriptive statistics regarding the number of animals enrolled (by season, by treatment group, and by dam’s parity), number of animals dead/euthanized, average body weight at enrollment, average rectal temperature at enrollment, and average dam’s gestation length by BRD status is presented in Table 1. No differences in the number of animals enrolled by season (*p* = 0.21), metaphylaxis treatment (*p* = 0.55), dam’s parity (*p* = 0.72), number of animals dead or euthanized (*p* = 0.73), average body weight at enrollment (*p* = 0.69), average dam’s gestation length (*p* = 0.98), or rectal temperature (*p* = 0.05) were observed between the BRD groups. However, the NBRD calves had a greater average daily gain during the pre-weaning period (*p* = 0.04) when compared to the EBRD and LBRD calves.

### 3.2. Association between BRD and Blood Chemical Panel Variables

**Figure 2:**
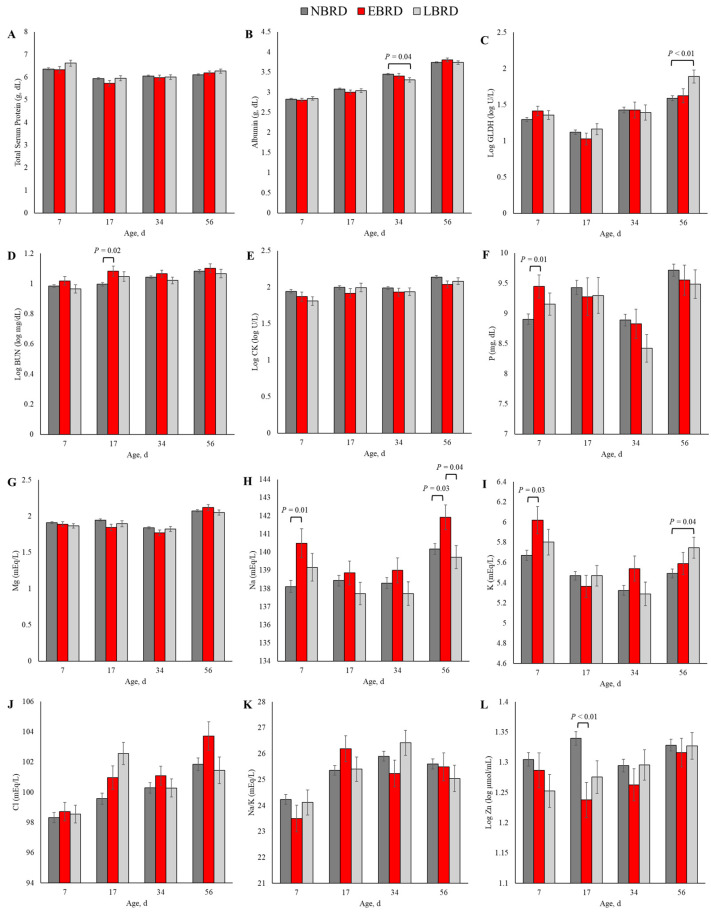
Least square means by days of sampling of (**A**) total protein, (**B**) albumin, (**C**) glutamate dehydrogenase (GLDH), (**D**) blood urea nitrogen (BUN), (**E**) creatine kinase (CK), (**F**) phosphorus (P), (**G**) magnesium (Mg), (**H**) sodium (Na), (**I**) potassium (K), (**J**) chloride (Cl), (**K**) sodium to potassium ratio (Na/K), and (**L**) zinc (Zn) by bovine respiratory disease (BRD) status (NBRD = no BRD; EBRD = BRD diagnosed ≤30 days of age; LBRD = BRD diagnosed >30 days of age). Error bars represent SEM. Bovine respiratory disease was diagnosed based on a systematic scoring system [4] based on the presence of nasal and ocular discharge, abnormal respiration, cough, ear droop or head tilt, and rectal temperature ≥39.2 °C.

The overall concentration of metabolites and minerals by BRD status is presented in Table 2. The concentrations throughout the study of CK (*p* = 0.01), Na (*p* < 0.01), Cl (*p* < 0.01), and Zn (*p* = 0.01) differed by BRD status. Additionally, the association of BRD status with GLDH (*p* = 0.04), P (*p* = 0.04), K (*p* = 0.04), and Cl (*p* < 0.01) were dependent on the day of sampling. The dynamics of the concentration of blood chemical panel variables in which the main effect of BRD status or the interaction term between BRD status and day of sampling yielded *p* ≤ 0.10 are illustrated in Figure 2.

Although we observed a *p* ≤ 0.10 for the interaction term between BRD status and time in linear models assessing the association of the blood concentration of the total protein (*p* = 0.07, Figure 2A), and Na/K (*p* = 0.08; Figure 2K), we did not observe any statistically significant differences in those variables based on BRD status in any timepoint of sampling. At 34 days of age, the concentration of albumin was 0.14 g/dL lower for the LBRD in comparison to the NBRD calves (*p* = 0.04; Figure 2B). The concentration of GLDH was 38.9 greater (back-transformed) for the LBRD calves compared to their NBRD (*p* < 0.01) counterparts at 56 days of age (Figure 2C). At 17 days of age, the EBRD calves had a 0.81 mg/dL (back-transformed) greater BUN concentration than the NBRD calves (*p* = 0.03; Figure 2D). Although the CK concentration was greater for the NBRD calves throughout the pre-weaning period (*p* < 0.01), no statistical differences were observed at any specific time point (Figure 2E).

At 7 days of age, the concentration of P for the EBRD calves was 0.54 mg/dL greater than for the NBRD (*p* = 0.01, Figure 2F). Although the main effect of BRD yielded *p* = 0.07 in the linear model assessing the association of Mg and BRD status, the concentration of this mineral and BRD status was not statistically significant at any of the time points evaluated (Figure 2G). The concentrations of Na (Figure 2H) and K (Figure 2I) differed by BRD status at two time points. Calves in the EBRD group had 2.38 and 1.76 mEq/L more Na in their serum than NBRD calves at 7 (*p* = 0.01) and 56 days of age (*p* = 0.03), respectively. Also, at 56 days of age, the concentration of Na was 2.21 mEq/L greater for the EBRD than the LBRD calves (*p* = 0.04). For K, the concentration was 0.35 mEq/L greater at 7 days for the EBRD than for the NBRD calves (*p* = 0.03), and 0.16 mEq/L greater for the LBRD than for their NBRD counterparts (*p* = 0.04). The concentration of Cl was 2.98 mEq/L greater for the LBRD than the NBRD at 17 days of age (*p* < 0.01, Figure 2J). The Zn concentration was lower at 17 days of age for calves with BRD (Figure 2L). In comparison to the NBRD, the Zn concentration at 17 days was 4.56 µmol/mL (back-transformed) lower for the EBRD (*p* < 0.01).

Neither the incidence of BRD nor the interaction term BRD*time were associated with the serum concentrations of glucose, bilirubin, creatinine, AST, globulins, albumin to globulin ratio, GGT, insulin, insulin to glucose ratio, Ca, and Mg, (*p* > 0.10).

### 3.3. Association of BRD with Acute Phase Proteins and Leukocyte Counts

The associations of BRD during the pre-weaning period with circulating concentrations of SAA and Hp and leukocyte counts are presented on Table 3. The serum concentration of SAA throughout the study period was associated with BRD status (*p* = 0.02). Additionally, the association of Hp with BRD status was dependent on the time of blood sampling (*p* < 0.01). Among variables related to leukocyte counts, monocyte counts were the only variable associated with BRD status (*p* < 0.01). The counts of white blood cells, neutrophils, lymphocytes, and neutrophil to lymphocyte ratio did not differ by BRD status, nor were they associated with the BRD status* time interaction term (*p* > 0.10). The dynamics of the concentrations of SAA, Hp, and monocytes count are illustrated in Figure 2.

**Figure 3:**
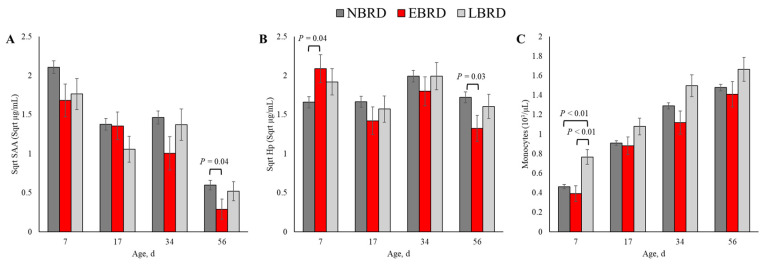
Least square means by days of sampling of (**A**) circulating concentration of serum amyloid A (SAA), (**B**) serum concentration of haptoglobin (Hp), and (**C**) monocyte counts by bovine respiratory disease (BRD) status (NBRD = no BRD; EBRD = BRD diagnosed ≤30 days of age; LBRD = BRD diagnosed >30 days of age). Error bars represent SEM. Bovine respiratory disease was diagnosed based on a systematic scoring system [4] based on the presence of nasal and ocular discharge, abnormal respiration, cough, ear droop or head tilt, and rectal temperature ≥39.2 °C.

The blood concentration of SAA was associated with BRD status at 56 days of age (Figure 3A). The SAA concentration was 0.27 µg/mL (back-transformed) lower for the EBRD calves than their NBRD counterparts at 56 (*p* = 0.04) days of age. In comparison to the NBRD, the EBRD calves had an Hp serum concentration 2.75 µg/mL greater at 7 days of age (*p* = 0.01), and 1.21 µg/mL lower at 56 days of age (*p* = 0.03; Figure 3B). At 7 days of age, the LBRD calves had 0.30 × 10^3^ and 0.37 × 10^3^ greater monocytes than the NBRD and EBRD calves, respectively (*p* < 0.01, Figure 3C).

## 4. Discussion

Transportation to an off-site calf raising facility has become a common practice in the North American dairy industry [9]. Transportation may be a stressor to young dairy calves that can further depress their immune system, which is not yet fully developed [6]. The risk of BRD and other diseases increases when stressors like transportation and comingling occur [6]. Previous studies have evaluated the association of the BRD and hematological variables [21,22,23], but few have looked at profiles following transportation stress [24]. Therefore, our objective was to investigate the association between BRD and hematological variables in a population of calves that underwent transportation stress during their first week of life.

The incidence of BRD in our study was 11.1%, which is lower than we had anticipated, especially because of the added stress of transportation during early life. Recent studies have reported BRD incidences of over 20% [25,26], with others reporting a BRD incidence as high as 63% [8]. Hence, our findings should be interpreted in the context of this study being conducted in a herd with a low BRD incidence. Also, it is important to highlight that this study utilized data from a subset of calves enrolled in a clinical trial designed to evaluate the effect of metaphylactic strategies against BRD in pre-weaned calves [19]. Blood samples were collected systematically at 7, 17, 35, and 56 days of age, and were not necessarily collected on the same day of BRD diagnosis. Hence, the concentration of blood biomarkers and leukocyte counts could have been assessed either before or after the BRD diagnosis. To address this issue, calves were categorized as NBRD, EBRD, and LBRD, so we could at least partially account for when BRD occurred during the pre-weaning period.

Complete blood cell count is an auxiliary exam that often aids in the diagnosis of diseases in cattle. For instance, it has been reported that BRD cases are followed by elevated WBC counts [27]. The only complete blood cell count variable associated with BRD status in this study was monocyte counts, which was elevated for LBRD calves at 7 and 34 days of age. However, others have not observed similar increases in monocytes related to BRD in beef calves [17] Other variables such as WBC, neutrophils, lymphocytes, and neutrophil to lymphocyte ratio were not different between BRD and non-BRD calves in the present study. We expected that the neutrophil to lymphocyte ratio would be associated with BRD in our calves, because it was found to be elevated in beef calves diagnosed with BRD [17]. Because increased neutrophil to lymphocyte ratio is a biomarker of stress [6], it is possible that transportation stress may have blunted the differences in the blood cell counts due to BRD status in our study.

Haptoglobin is an acute phase protein that is released by the liver in response to an inflammatory process that has been suggested as an important biomarker of disease in cattle [16,18]. Generally, greater circulating Hp concentrations is associated with BRD [26]. In our study, the association between Hp and BRD was dependent on the time of Hp assessment. While Hp was greater for EBRD than for NBRD calves at 7 days of age, this relationship changed at 56 days of age, with NBRD calves having greater Hp concentrations than their EBRD counterparts. No differences between LBRD and NBRD were observed. The findings from 7 days of age reflect the greater inflammatory status of calves that were likely experiencing BRD at the beginning of the pre-weaning period. Additionally, at 7 days of age, the calves had just undergone transportation stress a few days prior to Hp assessment. The blood concentration of acute phase proteins increases due to stressful events such as transportation [28,29], comingling [30], and sudden weaning [31]. Therefore, it is possible that the association between Hp at 7 days of life and BRD is related to calves with a lesser capability to cope with transportation and comingling while they were travelling to the calf raising facility. However, we have previously reported that a lower circulating concentration of Hp assessed at arrival was associated with a greater risk of BRD in calves transported to a calf raising facility [24]. Additionally, others observed that Hp was reduced following transportation [32]. Additionally, the findings at 56 days of age are puzzling, as calves that experienced BRD earlier in life had a lower Hp concentration than their NBRD counterparts. Similarly, the EBRD calves had a lower SAA concentration than the NBRD calves at the last sampling time point, also indicating that they were in a lower inflammatory state than calves that did not experience BRD in the pre-weaning period. 

Conversely, an increased serum concentration of SAA has been associated with BRD in calves [6]. Serum amyloid A is a better indicator of physical stress than Hp, and could represent a better biomarker for BRD for calves that undergo transportation stress. Perhaps some of the NBRD calves had subclinical pulmonary inflammation, which could explain this increase in acute phase proteins toward the end of the pre-weaning period. We acknowledge that measuring the Hp-matrix metalloproteinase 9 complex could have aided in detecting subclinical pulmonary inflammation, as its concentration has been considered a useful diagnostic tool after calves are challenged with *Bibersteinia trehalosi* and *M. haemolytica* [33]. Another biomarker of inflammation assessed herein is GLDH, which is an indicator of a systemic inflammatory response or liver damage [34,35]. For instance, increased GLDH was linked to hepatic lipidosis observed in cows with displaced abomasum [36]. In our study, we observed that in comparison to their NBRD counterparts, the LBRD calves had a greater GLDH concentration at 56 days of age, indicating that GLDH could also aid in the detection of BRD, at least when assessed later in the pre-weaning period. However, it was reported that GLDH decreased after an *Eimeria alabamensis* experimental challenge in calves, indicating that GLDH increases may not always be indicative of an inflammatory response in the gastrointestinal tract [37]. Also, this higher inflammatory state due to BRD incidence may be suggested by the lower albumin concentration at 34 days of life observed in the LBRD calves in comparison to their NBRD counterparts, as low levels of albumin have been associated with inflammation [38].

Herein, we observed that at 17 d of age, calves that experienced BRD within the first 30 days of life had elevated BUN in comparison to their healthy counterparts. This relationship between the BUN and BRD incidence in calves is challenged by previous reports. For instance, in one report, the BUN concentration was not associated with BRD in calves [27]. Another study demonstrated that BUN was negatively associated with the BRD incidence in high-risk beef stocker calves [39]. The dynamics of BUN in calves after a respiratory challenge with *Mycoplasma bovis* has indicated that the concentration of this biomarker can vary depending on the stage of infection, as it decreased between 0- and 7-days post-challenge, but increased between 7 and 14 days [40]. The added stress of transportation may have also contributed to our findings here. Previous findings suggest that BUN increases in stressful conditions due to compromised renal function [41,42,43]. Additionally, BUN is elevated during transportation in response to muscle protein breakdown that is caused by limited access to feed and water [6]. Perhaps, in our study, BRD calves had an increase in protein turnover during the time of disease, leading to the observed increase in BUN concentration in comparison to their healthy counterparts.

Creatine kinase is also a biomarker of muscle damage in cattle [44]. In the present study, NBRD calves had a greater CK concentration than calves diagnosed with BRD, although no statistically significant differences were observed on specific days of sampling. Elevated CK is often associated with increased muscle usage [45]. Although our calves were raised in individual hutches, and their physical activity may already be limited, it is important to highlight that calves diagnosed with BRD have decreased physical activity in comparison to healthy calves, marked by fewer step counts and more lying bouts, and increased lying times [46]. Hence, we could speculate that the decreased CK concentration observed in BRD calves is a consequence of the depressed behavior caused by the disease.

It has been reported that Ca, Mg, Na, K, and Cl are the main minerals affected during cattle transportation [6], but how these minerals are associated with the BRD incidence in calves submitted to long distance transportation within the first few days of life has not been previously assessed. We initially hypothesized that Ca would be an important mineral associated with BRD because Ca plays an important role in the immune system as it is utilized by immune cells during an inflammatory response [47,48]. However, we did not observe an association between Ca and BRD in our study. A decreased calcium concentration has been previously associated with infectious diseases in dairy cattle [49,50], which may indicate that a low Ca concentration may lead to increased susceptibility to infectious agents. However, this was not the case in our study participants.

Zinc concentrations were lower at 17 days of age in EBRD calves when compared to their healthy counterparts. This relationship between a decreased circulating Zn concentration during inflammatory responses has been previously reported. For instance, cows with mastitis had a lower blood Zn concentration than healthy cows [51]. Moreover, cows with uterine infections and inflammation after calving had decreased serum concentrations of Zn in comparison to cows without uterine infections [52]. It is likely that the same Zn response to infection and inflammation is experienced by young calves, as a decrease in circulating Zn concentration was observed when pre-weaned calves were submitted to an intravenous challenge with *E. coli* [53]. This reduction in circulating Zn may be at least partially explained by the Zn sequestration mechanism, which is part of the host response to a pathogenic challenge that reduces the availability of Zn to pathogens while prioritizing the use of Zn by immune cells [54].

We also observed that the electrolytes Na and K were associated with BRD, at least at one time point. Also, the Cl concentration throughout the pre-weaning period was greater for EBRD calves. The observed higher levels of these minerals in the blood may be indicative of dehydration. For instance, the Na concentration is typically affected by diarrhea in calves [55], but information about the relationship between BRD and the serum Na, K, and Cl concentrations is scarce. Additionally, the EBRD calves had a greater concentration of P at 7 days of age than the NBRD calves, a finding that can also suggest that BRD in calves may be linked to some level of dehydration [44].

The incidence of BRD was not associated with serum glucose, bilirubin, creatinine, AST, globulins, albumin to globulin ratio, GGT, Na to K ratio, insulin, or insulin to glucose ratio in the present study. We hypothesized that the incidence of BRD would be associated with glucose concentration, as it is known to decrease during an inflammatory response to a pathogenic challenge [53]. Perhaps we did not observe this same association between disease and glucose concentration because this biomarker is also affected by transportation stress, which was also experienced by our calves. For instance, transportation stress was followed by an increase in glucose concentration in the blood of bulls [56].

## 5. Conclusions

In conclusion, BRD status was associated with multiple hematological variables of calves that were subjected to long distance transportation during the first few days of life. Blood urea nitrogen, albumin, GLDH, CK, P, Na, K, Cl, Zn, Hp, SAA, and monocyte counts were associated with BRD status throughout or at least at one of the time points evaluated in this study. Glucose, bilirubin, creatinine, AST, globulins, albumin:globulin ratio, GGT, sodium:potassium ratio, Mg, insulin, and insulin:glucose ratio were also not associated with BRD status. Further studies are needed to assess the diagnostic and prognostic value of these variables associated with BRD in calves subjected to long distance transportation.

## Figures and Tables

**Table 1:** Descriptive statistics of bovine respiratory disease (BRD) categories.

Variable	BRD Group			*p*
	NBRD ^1^	EBRD ^2^	LBRD ^3^	
Total number of animals enrolled (%)	271 (88.9)	16 (5.2)	18 (5.9)	
Number of animals enrolled in spring (%)	169 (89.4)	7 (3.7)	13 (6.88)	0.21
Number of animals enrolled in winter (%)	102 (87.9)	9 (7.8)	5 (4.3)	
Number of animals enrolled in each metaphylaxis strategy (%)				
CON ^4^	89 (89.0)	3 (3.0)	8 (8.0)	0.55
META1 ^5^	93 (90.3)	6 (5.8)	4 (3.9)	
META2 ^6^	89 (87.3)	7 (6.9)	6 (5.9)	
Number of animals enrolled by dam’s parity (%)				
Parity = 1	31 (86.1)	3 (8.3)	2 (5.6)	0.72
Parity = 2	146 (88.0)	8 (4.2)	12 (7.2)	
Parity > 2	94 (91.3)	5 (4.8)	4 (3.9)	
Number of animals dead/euthanized (%)	5 (1.6)	0 (0.0)	0 (0.0)	0.73
Average body weight at enrollment, kg (SD)	32.9 (4.2)	32.0 (4.3)	32.6 (3.5)	0.69
Average rectal temperature at enrollment, °C (SD)	38.7 (0.3)	38.9 (0.3)	38.9 (0.3)	0.05
Average dam’s gestation length, d (SD)	278.5 (6.8)	276.5 (5.5)	277.6 (5.0)	0.98
Average daily gain during the pre-weaning period, kg (SD)	0.52 (0.1)	0.48 (0.1)	0.47 (0.1)	0.04

^1^ Calves not diagnosed with BRD; ^2^ calves diagnosed with BRD within the first 30 days of life; ^3^ calves diagnosed with BRD between the 31 and 56 days of life; ^4^ untreated controls; ^5^ single subcutaneous injection of tildipirosin (4 mg/kg of body weight) at enrollment; ^6^ subcutaneous injections of tildipirosin (4 mg/kg of body weight) at enrollment and 17 days later.

**Table 2:** Least square means of several variables related to metabolism and minerals measured in the serum of dairy calves during the pre-weaning period by bovine respiratory disease (BRD) status (NBRD = no BRD; EBRD = BRD diagnosed ≤30 days of age; LBRD = BRD diagnosed >30 days of age). Bovine respiratory disease was diagnosed based on a systematic scoring system [4] based on the presence of nasal and ocular discharge, abnormal respiration, cough, ear droop or head tilt, and rectal temperature ≥39.2 °C.

Variable	BRD			*p*		
	NBRD	EBRD	LBRD	BRD	Time	BRD*Time
Total Protein (g/dL)	6.12	6.06	6.21	0.40	<0.01	0.07
Albumin (g/dL)	3.27	3.25	3.23	0.44	<0.01	0.06
Glucose (mg/dL)	113.41	111.98	111.55	0.75	<0.01	0.69
Log GLDH ^6^ (log U/L)	1.36	1.37	1.45	0.19	<0.01	0.04
Log BUN ^1^ (log mg/dL)	1.03	1.07	1.02	0.05	<0.01	0.35
Creatinine (mg/dL)	0.81	0.79	0.78	0.49	<0.01	0.73
Sqrt Bilirubin (sqrt mg/dL)	0.45	0.44	0.47	0.18	<0.01	0.32
Log CK ^2^ (log U/L)	2.02	1.94	1.96	0.01	<0.01	0.80
Log AST ^3^ (log U/L)	1.63	1.62	1.62	0.63	<0.01	0.19
Globulins (g/dL)	2.88	2.85	3.02	0.23	<0.01	0.25
Log A/G ^4^	0.07	0.06	0.04	0.20	<0.01	0.97
Log GGT ^5^(log U/L)	1.84	1.82	1.90	0.24	<0.01	0.63
Log Insulin (log ng/mL)	1.52	1.52	1.57	0.77	<0.01	0.26
Log Insulin/Glucose	−0.52	−0.51	−0.47	0.64	<0.01	0.28
Calcium (mg/dL)	10.68	10.62	10.68	0.80	<0.01	0.17
Phosphorus (mg/dL)	9.23	9.27	9.09	0.55	<0.01	0.04
Magnesium (mEq/L)	1.94	1.91	1.91	0.07	<0.01	0.18
Sodium (mEq/L)	138.75	140.06	138.58	<0.01	<0.01	0.13
Potassium (mEq/L)	5.49	5.63	5.58	0.08	<0.01	0.03
Chloride (mEq/L)	100.01	101.13	100.71	<0.01	<0.01	<0.01
Na/K ^7^ (mEq/L)	25.27	25.10	25.25	0.86	<0.01	0.08
Log Zinc (log µmol/mL)	1.32	1.27	1.29	0.01	0.01	0.06

^1^ BUN: blood urea nitrogen; ^2^ CK: creatine kinase; ^3^ AST: aspartate aminotransferase; ^4^ A/G: albumin to globulin ratio; ^5^ GGT: gamma-glutamyl transferase; ^6^ GLDH: glutamate dehydrogenase; ^7^ Na/K: sodium to potassium ratio.

**Table 3:** Least square means of acute phase proteins and leukocyte counts in dairy calves during the pre-weaning period by bovine respiratory disease (BRD) status (NBRD = no BRD; EBRD = BRD diagnosed ≤30 days of age; LBRD = BRD diagnosed >30 days of age). Bovine respiratory disease was diagnosed based on a systematic scoring system [4] based on the presence of nasal and ocular discharge, abnormal respiration, cough, ear droop or head tilt, and rectal temperature ≥39.2 °C.

Variable	BRD			*P*		
	NBRD	EBRD	LBRD	BRD	Time	BRD*Time
Sqrt SAA ^1^ (sqrt µg/mL)	1.39	1.08	1.18	0.02	<0.01	0.18
Log Hp ^2^ (log µg/mL)	1.76	1.66	1.77	0.57	<0.01	<0.01
White blood cells (10^3^/µL)	10.33	10.40	10.70	0.67	<0.01	0.89
Log Neutrophils (log 10^3^/µL)	0.47	0.50	0.47	0.50	<0.01	0.86
Monocytes (10^3^/µL)	1.03	0.95	1.25	<0.01	<0.01	0.76
Lymphocytes (10^3^/µL)	5.89	5.88	5.87	0.99	<0.01	0.69
Log Neutrophil:Lymphocyte	−0.29	−0.26	−0.29	0.55	<0.01	0.51

^1^ SAA: serum-amyloid A; ^2^ Hp: haptoglobin.

## Data Availability

Data not available due to participating farm restrictions.
